# Mr. Charles Mortimer Lewis

**Published:** 1910-09-24

**Authors:** 


### OBITUARY.

MR. CHARLES MORTIMER LEWIS has died at "Chasemoor," Hindhead, Haslemere. He took his diploma in 1887, and became a student at St. Thomas's Hospital; afterwards he held the appointments of house-surgeon at Hove Hospital, and senior house-surgeon at the Brighton, Hove, and Preston Dispensary. He was also medical referee to the Scottish Imperial Assurance Company.

